# Large B cell lymphoma presenting as an adnexal mass in an HIV positive patient: a case report

**DOI:** 10.11604/pamj.2019.33.290.19459

**Published:** 2019-08-07

**Authors:** Misai Hukuimwe, Asaph Ziruma, Muchabayiwa Francis Gidiri, Marshall Manase

**Affiliations:** 1 Department of Obstetrics and Gynaecology, College of Health Sciences, University of Zimbabwe, P.O. Box A178 Avondale, Harare, Zimbabwe

**Keywords:** HIV, ovarian, non-Hodgkins lymphoma

## Abstract

We present a 34-year-old HIV positive woman who presented with a 2-month history of abdominal pain, abdominal distension, night sweats and fever. She had a firm, immobile and irregular abdominopelvic mass of about 30 weeks uterine size. Investigations showed a haemoglobin of 6.5g/dl, (NR 12-14) cancer antigen 125 of 44U/ml (NR 0-35), serum beta human chorionic gonadotropin (HCG) of 0.258mIU/ml (NR 0-5) and alpha fetoprotein of 7ng/ml (NR <10). Her CD4 count was 63cells/mm^3^. At laparotomy there was a left ovarian mass and the rest of the abdomen and omentum looked grossly normal, leading to the conclusion that the primary was in the ovaries. A total abdominal hysterectomy, bilateral adnexectomy and infracolic omentectomy were done. Sigmoidectomy and Hartmann's procedure were also performed. Histology of the specimens showed a large B cell lymphoma. She has since been commenced on chemotherapy and antiretroviral therapy and has been doing well.

## Introduction

We report a rare case of non-Hodgkin's lymphoma that presented to us as an adnexal mass in a 34-year-old woman, recently diagnosed of HIV. The presence of a large tumor involving both ovaries with insignificant disease elsewhere in the abdomen and pelvis lead us to the conclusion that the primary was in the ovaries. Primary Ovarian Non-Hodgkin's Lymphoma (PONHL) is very rare because the ovary lacks lymphatics. PONHL can however arise from lymphocytes, surrounding blood vessels and the corpus luteum [1]. If malignancy is suspected, women with ovarian tumors have to be subjected to surgical exploration. This means, surgical treatment and morbidity associated with it is unavoidable in most women with ovarian non-Hodgkin's lymphoma. These patients would be better managed with chemotherapy, radiotherapy or immunotherapy [2, 3]. Surgery plays a minor role in selected patients with non-Hodgkin's lymphoma [4]. Nodal non-Hodgkin's lymphoma has been associated with HIV infection. In sub-Saharan Africa where HIV is highly endemic, clinicians should consider the diagnosis of PONHL in women who present with adnexal mass in the background of an HIV infection.

## Patient and observation

A 34-year-old recently diagnosed HIV positive para 4 was referred to us with a 2-month history of abdominal pain, abdominal distension and backache which had worsened 2 weeks prior to presentation. She also had pain on defecation, night sweats and fever. On examination she looked ill, in respiratory distress with a respiratory rate of 28 breaths per min. She was febrile with a temperature of 38.2^0^C and had unilateral leg swelling. The abdomen was asymmetrically distended with an abdominopelvic mass of about 30 weeks size. The mass was firm, immobile with an irregular surface not attached to overlying skin. There was no ascites and no hepatosplenomegaly. The cervix looked grossly normal but was displaced anteriorly. Digital rectal examination revealed a mass in the pouch of Douglas not involving the rectal mucosa. Examination of the other systems was normal.

Investigations showed a haemoglobin of 6.5g/dl (NR 12-14g/dl), a slightly elevated cancer antigen 125 of 44U/ml (NR 0-35), serum beta human chorionic gonadotropin (HCG) of 0.258mIU/ml (NR 0-5) and alpha fetoprotein of 7 ng/ml (NR <10). Her CD4 count was 63cells/mm^3^. Her renal and hepatic function tests were normal. An ultrasound scan showed a solid mass measuring 19.6cm x 12.5cm, posterior to the uterine fundus and extending to the left adnexa. The uterus and its endometrium were normal. Doppler ultrasound scan of the right lower limb showed no evidence of thrombosis and her chest X-ray showed perihilar lymphadenopathy. The patient was taken for surgical exploration and staging for ovarian malignancy. At laparotomy there was a left ovarian mass attached to a bulky uterus and sigmoid colon. The right ovary was enlarged to about 10cm diameter. The left fallopian tube and ovary could not be distinguished and separated from the tumour. The rest of the abdominal organs and omentum looked grossly normal. There was no ascites. Tumour was friable and was removed by blunt and sharp dissection. Total abdominal hysterectomy, bilateral adnexectomy and infracolic omentectomy were done. The general surgeons were present and they did a sigmoidectomy and a Hartmann's procedure. She had enlarged retroperitoneal lymph nodes which were not dissected to reduce intraoperative bleeding and postoperative morbidity. We had encountered significant bleeding from the friable tumor. Patient was sent to the high dependency unit where she recovered postoperatively.

Histology of the specimens showed a large B cell lymphoma. A haematologist consulted and peripheral blood film and bone marrow aspirate were done. Both results were normal, ruling out abnormal cells in both samples. She was started on chemotherapy one month after surgery and by the time of writing she had received one cycle of cyclophosphamide, doxorubicin and vincristine. She has also been initiated on highly active antiretroviral therapy.

## Discussion

Primary Ovarian Non-Hodgkin's Lymphoma (PONHL) is very rare because the ovary lacks lymphatic tissue. It has been suggested that the PONHL arises from lymphocytes in the ovaries, surrounding blood vessels and the corpus luteum [1]. The pathology of PONHL is usually diffuse large B-cell lymphoma, which was the case in our patient. Fox *et al.* proposed diagnostic criteria for PONHL [5]. The lymphoma should be clinically confined to the ovary and a complete investigation fails to reveal evidence of lymphoma elsewhere. However, a lymphoma can still be considered as primary if it has spread to immediately adjacent organs or lymph nodes. The peripheral blood and bone marrow should not contain any abnormal cells and if any extra ovarian lymphomatous lesions arise there should be several months between the appearance of ovarian and extra ovarian lymphomatous lesions [5]. Our patient had involvement of the adjacent structures, namely: involvement of the sigmoid colon and local pelvic nodes. There were no abnormal cells in the peripheral blood and bone marrow and no extra ovarian lesions. She had perihilar lymphadenopathy seen on chest X-ray, but clinically, these lesions were small and most likely metastatic disease from the ovaries. There was no histological confirmation of these chest lesions.

The patient was managed as a case of ovarian malignancy with optimal cytoreductive surgery. Due to the rarity of ovarian lymphoma, surgery was done using the usual approach for ovarian malignancy. If it was possible to confirm the lymphoma before surgery, then surgery would not be the primary treatment. Most patients with ovarian lymphoma would have to be subjected to the morbidity associated with surgery as a result of this uncertainty before surgery. There are no radiological features differentiating Non-Hodgkin's Lymphoma (NHL) from other ovarian malignancies. About 40-70% of PONHL are bilateral [6]. In a series, bilaterality, homogeneity, absence of ascites and tumour almost always >5cm at the time of diagnosis are features that were associated with primary ovarian lymphoma [7]. In our case, we did not have access to computerized tomography scan or magnetic resonance imaging scan for better imaging, but current guidelines recommend surgery as first line in the evaluation of an adnexal or ovarian mass suspected to be malignant.

There are no controlled trials to guide the management of PONHL due to the rarity of disease. Treatment is based on standard of care for nodal NHL and case reports and series. Chemotherapy is the primary mode of treatment, with cyclophosphamide, doxorubicin and vincristine (CHOP) being the preferred regimen. Other treatment modalities include biological therapy with rituximab, radiotherapy and stem cell transplantation. Survival ranges widely between 0-36% less than 3 years post therapy. Compared with nodal lymphomas, ovarian lymphomas tend to have poorer outcomes due to late diagnoses, a problem commonly seen with other types of ovarian malignancy [8]. Emergency surgery is recommended for acute abdomen either due to haemorrhage into the tumour, torsion or for intestinal obstruction which is mostly palliative [4]. There are questions of whether surgery increases morbidity or mortality, but there is paucity of data due to the rarity of PONHL. Current evidence points to late presentation due to non-specificity of symptoms, late diagnosis and tumour histological type and grade as poor prognostic factors [8].

Bangera *et al.* described a case of a patient with HIV who had PONHL. She had been managed as an emergency and had a laparotomy for intraperitoneal bleed. A highly friable right ovarian mass was found and hysterectomy and bilateral salpingoophorectomy was done. Patient came back with a recurrence day 17 post operatively and she refused further treatment [9]. The incidence of NHL in patients with HIV infection greatly exceeds that of the general population. This risk is related to the transforming effects of the retrovirus, the immunosuppresion and cytokine dysregulation that results from the disease and opportunistic infections with other lymphotropic viruses [10]. These patients usually present with advanced stage bulky disease and typically involve extranodal sites. Our patient was diagnosed with HIV of which she was started on HAART soon after her first chemotherapy cycle.

## Conclusion

Ovarian non-Hodgkin's lymphoma should always be considered in women who are HIV positive presenting with adnexal masses. Research needs to be done to enable histological confirmation using less invasive techniques in patients who are HIV positive and therefore at significant risk of having ovarian non-Hodgkin's lymphoma. This will go a long way in avoiding surgery and its associated morbidity in these patients who usually do better with other forms of treatment.

## Competing interests

The authors declare no competing interests.
